# Stakeholder views on informed consent models for future use of biological samples in Malawi and South Africa

**DOI:** 10.1186/s12910-023-00882-4

**Published:** 2023-01-19

**Authors:** Francis Masiye, Walter Jaoko, Stuart Rennie

**Affiliations:** 1 Malawi University of Science and Technology, Ndata Farm, Thyolo, Malawi; 2 Centre for Medical Ethics and Law, Stellenbosch University, Cape Town, South Africa; 3 University of Nairobi, Nairobi, Kenya; 4 University of North Carolina, Chapel Hill, North Carolina, USA

**Keywords:** Informed consent, Broad consent, Tiered consent, Specific consent, Focus group discussion and in-depth interview

## Abstract

**Background:**

Current advances in biomedical research have introduced new ethical challenges in obtaining informed consent in low and middle-income settings. For example, there are controversies about the use of broad consent in the collection of biological samples for use in future biomedical research. However, few studies have explored preferred informed consent models for future use of biological samples in Malawi and South Africa. Therefore, we conducted an empirical study to understand preferred consent models among key stakeholders in biomedical studies that involve collection of biological samples in Malawi and South Africa. The main objective of the study was to explore views of key stakeholders on current policies on informed consent in Malawi and South Africa.

**Methods:**

This was a qualitative study involving in-depth interviews and focus group discussions. Thirty-four in-depth interviews and 6 focus group discussions were conducted with REC members, Funders, Policymakers, CAB members and Research Participants in Malawi and South Africa to gather their views on models of informed consent. The study was conducted in Cape Town, South Africa, and Blantyre and Lilongwe in Malawi.

**Results:**

Most key stakeholders preferred broad consent and tiered consent to specific consent. Some participants expressed a strong preference for specific consent to other models of informed consent in biomedical research. Few participants did not have any preference for a consent model, opting for any consent model which provides adequate information about the proposed research and what their national consent regulations require. Finally, very few participants preferred blanket consent to other informed consent models.

**Conclusions:**

This study aimed to help fill the gap in the scientific literature on key stakeholder views on consent models for future use of biological samples in Malawi and South Africa. The findings of the study have provided some evidence that may support policies on permissible consent models for future use of biological samples in sub-Saharan Africa considering the differences in informed consent regulations and guidelines. Finally, the findings can inform ongoing discussions on permissible consent models to be used for future use of biological samples.

**Supplementary Information:**

The online version contains supplementary material available at 10.1186/s12910-023-00882-4.

## Background

There are controversies regarding the use of broad consent when collecting biological samples in biomedical research in both high income and low- and middle-income countries. Broad consent, as defined here, is a type of consent obtained at the time of recruitment into a study that allows researchers and other secondary users access to biological samples in current and all unspecified future research anytime and anywhere [1]. The recent South African Department of Health (DOH) research ethics regulations and guidelines allow researchers to obtain broad consent from research participants for future research purposes on condition that researchers apply for ethics review and approval of any future research [2]. These research ethics regulations and guidelines on broad consent and future use of biological samples and data collected in biomedical research are consistent with the vision of the H3Africa Initiative, which requires that consent should be broad enough to allow future and secondary uses of biological samples and data in advancing knowledge to improve health. The H3Africa Initiative is a consortium of African scientists funded by the Wellcome Trust and the US National Institutes of Health in partnership with the African Society for Human Genetics whose main aim is to foster genomic research expertise on the African continent with the goal of using genomic methods to address health inequities in both communicable and non-communicable diseases in Africa [3]. The main objective of the H3Africa consortium is to enhance the capacity of African researchers to conduct genomics research among African populations. Most H3Africa research grants are awarded directly to African institutions where principal investigators are based, and this allows African scientists to develop and direct their independent research agendas [3]. Recommendations from the H3Africa Consortium Ethics Consultation Meetings informed these recent South African research ethics regulations and guidelines on broad consent. However, in their paper, De Vries et al. reported that some key-stakeholders such as REC members and researchers are not clear on the conditions under which broad consent should be sought since there was no education of key-stakeholders about the new policies. The paper also reports that key-stakeholders were not consulted when the Department of Health in South Africa decided to come up with the current policies on broad consent and claim that the policies on broad consent were not evidence based since there were no studies conducted to explore key-stakeholder views on their preferred consent models in the South Africa context—the argument on the second point is that the policies on broad consent were not informed by evidence [4].

In contrast, research policies in Malawi do not allow researchers to obtain broad consent from research participants [5]. However, researchers who receive H3Africa funding are encouraged to obtain broad consent from research participants but this is not an official requirement by H3Africa [6]. Although this is not a requirement for funding, H3Africa is supportive of this policy as it will facilitate future research and it is consistent with current international research practices. This recommendation by the H3Africa has become a contentious issue among researchers, research ethics committee (REC) members and policymakers in Malawi [7]. To address these issues, some scientists recommended that empirical studies need to be conducted to provide evidence that would empower policymakers to make informed decisions about policies on acceptable consent models in biomedical research [4]. Hence, this study attempted to provide empirical evidence on acceptable consent models in biomedical research from key stakeholders with the goal of potentially influencing current policies on consent in Malawi and South Africa.

## Methods

### Study design

The study employed an in-depth exploratory study design to collect data from research participants. Both deductive and inductive approaches were used with the exploratory study design. The qualitative study approach enabled the researcher to derive in-depth information from study participants [8]. The study was descriptive and exploratory in nature as it attempted to understand stakeholder views on current consent models used in biomedical research and their preferred consent models.

### Data collection methods and study setting

In-depth interviews (IDIs) and focus group discussions (FGDs) were used to collect data from study participants in both Malawi and South Africa. The two qualitative data collection methods complemented each other. Issues that came out in the FGDs were explored further in the IDIs with respondents. This qualitative study approach allowed the investigators to derive in-depth information from study participants.

The two study contexts in sub-Saharan Africa, namely Malawi and South Africa, were chosen because they have strikingly different policies on consent in biomedical research. In Malawi, broad consent is not allowed under current regulations while in South Africa, broad consent is allowed and even preferred. These differences in policies provided rich data for comparison among stakeholders involved in clinical research in the two countries.

The study targeted key stakeholders in biomedical research in both countries. The stakeholders included policymakers in the Ministries/Departments of Health, funders of biomedical research, REC members, Community Advisory Board (CAB) members, Patient Advisory Group (PAG) members and research participants taking part in biomedical research. More specifically, in Malawi, the study recruited policymakers in the Ministry of Health (MOH) and the National Commission for Science and Technology (NCST), sponsors/funders of biomedical research, CAB members, PAG members; and research participants taking part in biomedical research. In South Africa, the study recruited policymakers in the Department of Health, funders of biomedical research, REC members, CAB members, PAG members and research participants taking part in biomedical research.

### Participant recruitment and enrolment

A purposeful sampling method was used to recruit all participants in this study. This sampling method was chosen because the study targeted key stakeholders that developed and implemented policies on consent in both countries. The targeted stakeholders had not participated in a study on this topic before and there was no study that had reported on the study topic in the two settings. An attempt was made to recruit a representative sample of both males and females, individuals from different ethnic groups, and of different ages in each category to adequately capture the heterogeneity among the key stakeholders. Two separate demographic data sheets were developed for both IDI respondents and FGD participants to document the sex, age, ethnicity, and highest attained educational qualification of each participant. These demographics were used in the analysis of data collected from the participants. Four (4) Directors of Research and members of staff involved in developing health research policies in the Ministries/Departments of Health in both Malawi and South Africa were recruited. Four (4) funders of biomedical research were enrolled in both countries. The study also recruited ten (10) REC members who review biomedical research studies in both countries. Thirty-four (34) IDIs were conducted with respondents in both countries. Six (6) FGDs were conducted with research participants taking part in biomedical research in both countries (three (3) FGDs in each country). Each FGD targeted a minimum of six (6) participants and a maximum of ten (10) participants. In total, forty (40) interviews (34 IDIs and 6 FGDs) were conducted with potential study participants in both countries. In total, a minimum of seventy (70) study participants and a maximum of ninety-four (94) study participants were recruited in both countries. Details about the total number of interviews and the categories of respondents recruited in each site are shown in Table 1.

**Table 1:** Interview types, numbers, and category of respondents per research site

Type of interview	Number of interviews	Category of respondents	Site
FGD	3	Research participants (25)	Malawi
FGD	3	Research participants (19)	South Africa
IDI	2	Policymakers	Malawi
IDI	2	Policymakers	South Africa
IDI	2	Funders	Malawi
IDI	2	Funders	South Africa
IDI	5	REC members	Malawi
IDI	5	REC members	South Africa
IDI	4	CAB members	Malawi
IDI	4	CAB members	South Africa
IDI	4	PAG members	Malawi
IDI	4	PAG members	South Africa
Total	40		

### Recruitment of study participants in Malawi

Research participants and CAB members were identified through the Malawi-Liverpool-Wellcome Trust. Members of PAG were identified through the Malawi Human Rights Watch and the Malawi Cancer Consortium. REC members were identified through two research ethics committees that review both biomedical and social science research namely the College of Medicine Research and Ethics Committee (CoMREC) and the Malawi University of Science and Technology Research Ethics Committee (MUSTREC). Researchers who conduct biomedical research whose health research studies were reviewed by the MUSTREC and CoMREC were identified by sponsors/funders of biomedical research. The funders of biomedical research recruited into the study included two major research funders in Malawi.

### Recruitment of study participants in South Africa

Research participants and CAB members were identified through the research centres and units based at the Stellenbosch University Faculty of Medicine and Health Sciences such as the Desmond Tutu TB Centre (DTTC), Family Clinical Research Unit (FAMCRU), African Cancer Institute, Centre for Tuberculosis Research and TREAD Research. Members of PAGs in South Africa were identified through the Centre for Medical Ethics and Law at Stellenbosch University. REC members were identified through the National Health Research Ethics Council (NHREC) and the targeted RECs were the 2 Stellenbosch University Health Research Ethics Committees (HRECs). Sponsors/funders of biomedical research recruited into the study included the Medical Research Council (MRC). The sponsors/funders of biomedical research were also identified through researchers based at the Stellenbosch University who were recruited into the study.

### Recruitment procedures in Malawi and South Africa

The principal investigator approached potential participants via emails and those who expressed their willingness to participate in the interviews were scheduled to take part in the in-depth interviews. Researchers who are conducting biomedical research in the two countries identified research participants who are participating in their biomedical research studies. Research staff who implement biomedical research protocols approached research participants and informed them that a new study was being conducted to understand participants’ preferences on consent models and future use of biological samples. Research participants who showed interest to participate in the new study were referred to the principal investigator who was available at the research site. The principal investigator explained details of the study to those who were interested, gave them copies of the study information sheet and scheduled them for FGDs on separate days. On the day of the FGD, the principal investigator or research assistant explained details of the study again using the information sheet for FGD participants and obtained written informed consent from each of the potential participants in the FGD before conducting the FGD. The research assistant facilitated the FGDs in English in South Africa while the principal investigator facilitated the FGDs in Chichewa in Malawi. Participants who took part in the IDIs were approached via emails. The email content introduced the study to them and highlighted that the student investigator was conducting a qualitative study on consent and future use of biological materials in Malawi and South Africa. It requested the potential participants who were willing to take part in the study to express their willingness to participate in the study in their response email. Those who expressed their willingness to participate in the interviews were asked to choose whether they wanted to be interviewed virtually or physically. The potential participants were then scheduled for the IDIs. Those who had chosen to be interviewed virtually received both a consent form to sign and an interview guide. They were asked to sign the consent form and return it to the principal investigator. For those who had chosen to be interviewed in person, they received a copy of the interview guide and written informed consent was sought from them on the day of the interview.

### Data collection methods

IDIs were conducted with policymakers, sponsors/funders, REC members, PAG members and CAB members in both countries while FGDs were conducted with research participants who are taking part in biomedical research in both Malawi and South Africa. The principal investigator used semi-structured interview guides to collect information from participants (Additional file 1). Each IDI took approximately 40 minutes. Each FGD took approximately 55 minutes and all FGDs were conducted physically at private places in both countries. COVID-19 preventive measures such as wearing of face masks, hand washing, sanitization and physical distancing were observed during both face-to-face FGDs and IDIs. Open-ended questions were used to guide the interviews. All participants agreed to be audio-recorded during the interviews.

Study participants in both IDIs and FGDs were asked to state their preferred model of consent among four models of consent consisting of broad consent, tiered/multi-layered consent, specific consent, and blanket consent. The four models of consent were explained to the study participants before they were asked to choose their preferred model of consent. Briefly, broad consent implied consent that allows researchers and other secondary users access to biological samples in current and all unspecified future research anytime and anywhere with conditions/restrictions such as ethics review of any future research by an independent Research Ethics Committee; tiered or multi-layered consent implied consent that provides research participants with several options for the use of their biological samples, for example, for the primary research only or for the primary research and some secondary research in a related field to the primary research; specific consent implied consent that allows researchers to collect biological samples from research participants and use them in the specific research for which they are collected only and does not allow any future or secondary use of biological samples and data outside the scope of the current study; and finally, blanket consent implied consent that allows researchers to collect biological samples from research participants and use them for both the current research and future research of any kind without restrictions.

### Data processing and management

All audio-recordings in the IDIs and FGDs were transcribed verbatim. The transcripts were coded according to the type(s) of interviews and the type of respondents who participated in the different interviews in both Malawi and South Africa. For example, an IDI conducted with a policymaker in Malawi was coded as MW_IDI_PM_01 while in IDI conducted with a policymaker in South Africa was coded as SA_IDI_PM_02. Similarly, a FGD conducted with research participants in Malawi was coded as MW_FGD_RP_01 while in South Africa, it was coded as SA_FGD_RP_02. All transcripts were saved on the laptop of the student investigator before each transcript was exported into Atlas.ti for data analysis.

### Data analysis

Data were analysed thematically. The data were entered into Atlas.ti for analysis. The analysis was iterative. The preliminary analysis of data at each stage of interviews informed interview questions for the subsequent interviews and by the time the final interviews were done, data saturation had been reached. A coding framework was developed after thorough reading of the transcripts, and it was further discussed and applied to the transcripts in Atlas.ti.

## Results

### Demographic characteristics of study participants

A total of 34 IDIs and 6 FGDs were conducted in Malawi and South Africa. Seventy-eight (78) participants took part in the IDIs (34) and FGDs (44). Most of the participants recruited into the study (47/78) were females and aged between 20 and 65 years. The demographic characteristics of the study participants are shown in Table 2.

**Table 2:** Demographic characteristics of study participants

Demographic characteristics	IDI respondents (N = 34)	FGD participants (N = 44)
*Gender*		
Male	13	18
Female	21	26
*Age*		
20–39	25	37
40–65	9	7
*First language*		
Afrikaans	16	17
Chichewa	14	25
English	4	2
*Highest level of education*		
Primary	3	12
Secondary	14	26
Tertiary	17	6
*Ethnicity*		
Black African	25	29
Coloured	5	13
White	4	2

#### Preference for broad consent and tiered//multi-layered consent

The main finding in this study was about participants’ preference for broad consent and tiered consent versus specific consent. Some participants had more than one preference. Most study participants in the IDIs (23/34) and FGDs (4/6) in Malawi and South Africa preferred broad consent and tiered consent to specific consent for use in clinical studies in which biological samples are collected. They were various reasons that were given for preferring broad consent and tiered consent to specific consent. Some of the reasons were that it would be very expensive to collect biological samples using specific consent and destroy left-over samples after the study is over and the value of keeping samples indefinitely for future studies. Thus, some of the participants said:

…As a researcher it’s not always possible to go for specific consent because it is extremely expensive to collect samples and data for a specific study and destroy them after the study. Because I am specifically thinking about my research …. So, I am guided through the literature, and I think it’s extremely expensive research and it’s extremely time consuming. So, if you are guided by the literature, I think it would be valuable to go for broad consent or tiered consent which will allow you to do future research using the available samples and data as long as you obtain HREC approval for any future research on the samples and data (SA-IDI-REC-001).

I prefer the last one (I: Broad consent) yes...I know that they don’t have to take another consent from me if they want to use the samples in future – they can use the samples in any future study (P5, SA-FGD-RP-03)

And another participant said;

…If you are working in the field of genetic research or that type of research where you are looking at different markers and DNA then it becomes almost narrow minded to look only at what you are doing right now without rethinking you know about what is going to happen in the future… because as you are doing everyone else is also doing research that informs future work. So, I can’t really say which one I would prefer but perhaps a combination of all of them (SA-IDI-REC-005)

Tiered consent and broad consent were rated at the same level by the participants. They highlighted that tiered consent gives options to study participants to choose whether they would want their samples stored for future research or not thereby giving them power to choose what happens to their biological samples. In tiered consent participants have an opportunity to specify a disease category in which their samples can be used in future. They also explained that both tiered and broad consent entail future use of biological samples. One of the participants said:

To be honest, I wouldn’t go for specific consent ...broad consent or tiered consent is better and the reason why I am saying this is because if all the information that is required is given in the broad or tiered consent form, I have the choice of making an informed decision because of the information that has been given to me. So, whether it is a broad consent or a tiered consent as long as the information is included in the consent, it wouldn’t really matter to me (MW-IDI-CAB-002).

### Preference for specific consent

On the contrary, some participants (12/34) preferred specific consent to broad consent and tiered consent. Some of the participants who preferred specific consent argued that potential participants are mostly approached to participate in specific studies. As such, these participants said researchers should not ask for broad consent nor tiered consent on account that they would not fully understand the nature of future studies at the time of providing consent. They also argued that specific consent would ensure that researchers do not abuse biological samples in future studies. Thus “...The reason why Malawi does not allow those other types of consent is just to safeguard the specimens that are obtained from our research participants so that researchers don’t abuse them anyhow” (MW-IDI-Policymaker-002).

Participants have to consent to something they fully know and understand. So, you can’t ask a participant to consent to future research which is not yet known. How can I consent to something l don’t know? That is not informed consent ...it is cheating participants and we cannot allow that in this country (MW-IDI-Policymaker-001)

Other research participants said they preferred specific consent to broad consent though research guidelines may allow broad consent because specific consent is easy to implement as it does not require any governance structures about the use of samples and data in future. They also observed that there are discrepancies in implementing the Department of Health (DOH) guidelines on consent. While some researchers understand the DOH guidelines on consent models, they prefer using specific consent because it is the only model of consent they are used to. Participants also observed that a study has a specific period and that consenting must be done every time a study is being carried out. So, specific consent has to be administered every time a study is being conducted on either stored samples or data.

… this is where there is a lot of discrepancy because not all researchers follow those DOH guidelines (about broad consent). I am not sure about other researchers but from what I know, some researchers prefer to use specific consent because it is not difficult to implement (SA-IDI-REC-03).

Hmmm I think most studies are very specific. They try to answer a specific question. So, I think specific consent is the best type of consent for biomedical studies since research subjects are asked to participate in specific studies (MW-IDI-REC-05).

As a patient advocate, I would go for specific consent for joining research because consent is given for participation in a study at a time. If a researcher intends to do multiple studies with my data or blood, he must obtain my consent every time he wants to do a study (SA-IDI-PAG-01).

### No specific preference for any consent model

Few participants (4/34) explained that they would not have any preference for a consent model. Rather it would depend on the information provided in the information leaflet about the study in question. They would choose the type of consent in which adequate information is provided to potential research participants about the study.

Thus,

To be honest, I wouldn’t actually have a specific preference and the reason why I am saying this is because if all the information that is required is in all those 4 consents that you mentioned then I have the choice of making an informed decision because of the information that has been given to me. So, whether it is a blanket consent or a tiered consent or the other 2 that you mentioned, as long as the information is included in the consent, it wouldn’t really matter to me (SA-IDI-CAB-003).

If I am not mistaken, when you are talking about the different models, you have different components of certain studies where you have the main consent, then you have the genetic consent, then you have the blood storage consent for when you are using samples like blood samples or sputum. In most cases, blood storage consent would be for bloods. Then you also have assent for minors under 18, because according to research ethics, minors have to sign assent as well as the main consent that is then signed by the legal parent or guardian. So, each component of the study has sometimes different consent forms, where in some studies you might have maybe just the main consent form … depending on what the nature of the study is (P8, MW-FGD-RP-03).

Among the few research participants, some indicated that researchers are obliged to follow research guidelines of a country where they live and conduct research. As such, no researcher would prefer a consent model other than following what the research regulations in each country say about the accepted model of consent in the country where the research is being conducted.

For example:

As funders of research, we do not dictate what model consent researchers we fund should follow. It all depends on what the national regulations require, and we do not interfere with such regulations in the countries where we fund researchers (MW-IDI-Funder-02).

We implement research regulations in our country when we review research proposals. So, we do not choose what type of consent our researchers ought to use. They are bound to follow what the NCST requires in terms of consent (MW-IDI-REC-02).

### Preference for blanket consent

Very few participants (3/34) preferred blanket consent to other consent models. The first reason provided for preferring blanket consent was that it allows biological samples to be used in any type of research without any restrictions. The second reason was that when one donates biological samples, he/she gives away his/her right to the samples. As such, the samples can be used in any future research without any strings attached. These same reasons were cited both by those who preferred blanket consent and those who did not.

Blanket consent is good because samples can be used in any type of research and there is value for money used to collect the samples (MW-IDI-PG-001)

…I would be very emphatic about reiterating to the participant when it comes to the blanket consent that the participation is voluntary and they can withdraw but they just need to be sure that they are giving blanket consent which means that even if a researcher comes 10 years later and wants to use my samples, I have given him or her that right to use them (SA-IDI-CAB-002).

The participants also emphasized the fact that participants have the right to choose any type of consent they prefer if they have been given adequate information about the type of consent they are being asked to provide.

I do not think blanket consent should be a deterrent for people wanting to give consent as long as the information is provided. If people were given that information that you have explained to me, they will decide if they want to give blanket consent (MW-IDI-CAB-01).

### Discussion of findings

It is very interesting to note that the preference of broad consent to specific consent in this study is consistent with the vision of the H3Africa, and the consent policies of some Sub-Saharan African countries such as South Africa, Nigeria, and Cameroon [9]. Both the H3Africa and the consent policies of the above named Sub-Saharan African countries allow researchers to obtain broad consent for the use of biological samples in future research [9]. This finding is also consistent with the 2018 revisions to the Common Rule also known as the US Federal Policy for the Protection of Human Subjects issued by the US Department of Health and Human Services (DHHS) that were effective in January 2019 which introduced broad consent. The revision to the Common Rule introduced the third option of broad consent. According to the revised Common Rule, broad consent can only be used to obtain a study participant’s consent for the storage, maintenance, and secondary research use of identifiable private information or identifiable biological samples [10]. All researchers who receive US federal funding are required to abide by the Common Rule in the conduct of their human subjects’ research and researchers in Malawi and South Africa who receive such funding are no exception. However, unlike blanket consent, broad consent comes with conditions/restrictions such as ethics review of any future research by an independent Research Ethics Committee; the right for research participants to withdraw from research participation; the requirement for community engagement; and the requirement for clear access and use policy frameworks for future research [3]. It is also argued that broad consent requires good governance linkages to ensure that biological samples that are used for future research are within the specific areas or categories of research to which participants consented and the future studies comply with the restrictions stipulated above. Proponents of the broad consent model argue that broad consent is consistent with current international research practice; the benefit is high in that it allows potentially fruitful and important future research to be conducted and it respects participants’ autonomy to participate in future research even though they may not understand its full scope since it may be impossible to stipulate all future research uses [1, 11, 12]. While it is important to consider the rights of individuals who provide biological samples, it is undeniable that future use of biological samples maximizes the value of biological samples obtained in research and helps to promote societal good. According to the World Medical Association Declaration of Taipei, there is always a need to achieve a balance between the rights of individuals giving their tissue or data for research and other purposes based on confidentiality and privacy rules while at the same time recognising that health data has become a very powerful tool for increasing knowledge [13]. On the other hand, others have argued that broad consent breaches on the essential elements of an informed consent since participants do not understand fully all future aspects of a research project; it is difficult for participants to withdraw consent when they do not know the future studies they will participate in; and it may be impossible for participants to know future study risks of harm [14–16]. Milkensen and others also argue that broad consent does not provide adequate protection as it fails to satisfy both the value criterion and the duration criterion and they propose two elements that ensure that broad consent is deep [17]. The elements they propose for ensuring that broad consent provides adequate ethical protection to research participants are ensuring a strong and continuous ethical review process of any future research and continuous provision of information to participants [17].

Despite the arguments for and against broad consent explained above, defining informed consent requirements for collecting, storing, and using biological samples for research remains a controversial international issue [1, 18]. While some high income countries support broad consent, some studies suggest that some research communities in low- and middle-income countries have not convincingly embraced broad consent, and they question the appropriateness of applying the informed consent requirements of high income countries such as the USA and UK to manage the use of biological samples in low- and middle-income countries, including those in Sub-Saharan Africa [19–22]. These controversies became louder with the establishment of the H3Africa Consortium where scientists and researchers are required to obtain broad consent from research participants for future use of biological samples collected in genomic studies funded by the NIH and the Wellcome Trust. Consistent with the vision of the H3Africa, some Sub-Saharan African countries such as South Africa, Nigeria and Cameroon allow researchers to obtain broad consent for the use of biological samples in future research. On the contrary, other Sub-Saharan African countries such as Malawi, Zambia and Tanzania do not allow researchers to obtain broad consent for the use of biological samples in future research [9]. The preference of tiered or multi-layered consent to specific consent is consistent with a study conducted with South African research participants. In this study, most participants preferred tiered consent to other models of consent because it gave them options regarding future use of their biological samples and they felt it was more consistent with the principle of respect for autonomy though a few of the participants in the same study expressed the desire to be re-contacted for consent for future use of their biological samples [18]. In similar studies conducted in Egypt and Nigeria, 44% and 25% of the participants in Egypt preferred tiered consent in Egypt and Nigeria, respectively [21, 22]. Tiered consent is also currently allowed in Botswana, Sierra Leone, Senegal, and Uganda [9].

On the contrary, some participants in our study preferred specific consent to broad consent. Specific consent is the most common type of consent that is used in biomedical research. Specific consent allows researchers to collect biological samples from research participants and use them in the specific research for which they are collected. It does not allow any future or secondary use of biological samples outside the scope of the current study. It requires researchers to re-consent research participants for new use of their biological samples that is outside the scope of the original consent. This finding is consistent with consent models used in Malawi, Zambia, and Tanzania among other countries [9].

There are several disadvantages of this consent model. Firstly, it is very difficult to trace study participants later after the original study is over. This results in a lot of waste in biological specimens since they cannot be used for advancement of scientific knowledge. Secondly, regulations in some countries require that any left-over samples from the original study must be destroyed since they cannot be used in future research. Destruction of biological samples limits opportunities for promotion of potentially beneficial future biomedical research.

With regards to preference for blanket consent, some have argued that blanket consent is not informed consent at all since participants are not specifically informed about the type of future use of biological samples and their consent allows an unlimited range of options. They also argue that any future research using biological samples that are collected with this consent model becomes unethical since it does not require ethics approval. We agree with these sentiments that blanket consent is not informed consent at all because participants cannot agree to something they do not know. Knowledge and understanding of what happens to one’s biological samples is very critical to any informed consent. Therefore, it is not surprising that blanket consent is not allowed in Sub-Saharan Africa [9].

Here it is worth to note that the strength of this study was that it sought participants’ opinions on all the four models of consent. As far as we know, this was the first study in sub-Saharan Africa to explore participants’ views on all the four consent models.

We are aware that there are different regulations and guidelines on models of permissible informed consent in sub-Saharan Africa and we hope that this study has provided some information that may inform policies on permissible consent models in sub-Saharan Africa.

## Conclusions

This empirical study has attempted to fill the gap in literature on key-stakeholder views on consent models for future use of biological samples in Malawi and South Africa.

The findings of this study may influence policies that govern health research in Malawi and South Africa including similar settings. Specifically, the findings of this empirical study provide some evidence to support policies on consent and future use of biological samples in both countries. Findings of the study may also inform ongoing discussions on appropriate consent models to be used for future use of biological samples.

## Supplementary Information

**Additional file 1:** Semi-structured interview guides (FGD and IDI guides) for the study.

## Data Availability

The datasets used and/or analysed during the current study are available from the corresponding author on reasonable request.

## Abbreviations

CAB: Community Advisory Board
COMREC: College of Medicine Research Ethics Committee
DNA: Deoxyribo-nucleic acid
DOH: Department of Health
DTTC: Desmond Tutu TB Centre
FAMCRU: Family Clinical Research Unit
FGD: Focus Group Discussion
HREC: Health Research Ethics Committee
IDI: In-Depth Interviews
MOH: Ministry of Health
MRC: Medical Research Council
MUSTREC: Malawi University of Science and Technology Research Ethics Committee
NCST: National Commission for Science and Technology
NHREC: National Health Research Ethics Council
NIH: National Institutes of Health
PAG: Patient advocacy group
REC: Research Ethics Committee
UNC: University of North Carolina
