# Supplementary material for: Stakeholder views on informed consent models for future use of biological samples in Malawi and South Africa
Source: BMC Med Ethics. 2023 Jan 19;24:4. doi: 10.1186/s12910-023-00882-4 (PMC9854061; doi:10.1186/s12910-023-00882-4)
Supplement: Supplementary file 1 — Additional file 1: Semi-structured interview guides (FGD and IDI guides) for the study. [file 12910_2023_882_MOESM1_ESM.pdf]

APPENDIX E: IN-DEPTH INTERVIEW GUIDE FOR REC MEMBERS, CAB MEMBERS AND PAG MEMBERS

Title of the study: Stakeholder views on current policies on consent and future use of biological samples and data collected in biomedical studies in Malawi and South Africa

Principal Investigator: [redacted]

Co-Investigators: [redacted]

1. **Knowledge about types/models of consent used in biomedical research**
    a. What does the word "consent" mean for you?
    b. Why do researchers obtain consent from research participants?
    c. What types/models of consent do you know? (Explain the four models of consent to respondents) what she/he understands about the four different types/models of consent)
    d. Which type/model of consent do you prefer and why do you prefer this type/model of consent?
2. **Knowledge and understanding of national regulations and guidelines on informed consent in biomedical research**
    a. Why do you think this country requires researchers to seek consent before enrolling participants in biomedical research?
    b. Do you know what the regulation says about seeking consent from research participants? If yes, please, tell me about any national regulation or guideline on informed consent that you know. If the answer is no, the student researcher will explain the current national regulation or guideline on consent in biomedical research to respondents. (Probe: what do you think were the reasons why the policy-makers decided to come up with such a regulation or guideline on informed consent?)
    c. What is your opinion on this regulation/guideline? (Probe: Why or why not?)
    d. What do you think are the problems or challenges of such regulation? (Probe: What changes if any should be made on this regulation?)
    e. What is your opinion on obtaining broad consent from research participants? (Probe: what are the opinions of other researchers/REC members on broad consent?)

3. **<u>Knowledge and understanding of national regulations and guidelines on use of biological samples and data collected in biomedical research</u>**

a. Do you know why researchers obtain samples such as blood from research participants? If yes, why do you think they obtain such samples? If no, the student will explain what the samples are used for.

b. Do you know what the country requires researchers to do with the samples they obtain from research participants? If the answer is no, explain the current national regulation or guideline on use of biological samples and data collected in biomedical research to respondents) (If the answer is yes, please explain the regulations or guidelines you know on the use of biological samples and data collected in biomedical research)

c. For how long do the regulations or guidelines allow biological samples and data collected in biomedical research to be stored? (Probe: why do you think the policy-makers decided to come up with such a regulation/guideline? What is your opinion about it?)

d. Do the national regulations or guidelines allow future use of biological samples and data collected in biomedical research? (Probe: why/why not? What is your opinion on this?)

e. What do you think about the secondary use of biological samples and data collected in biomedical research in future unspecified research?

f. In your opinion, who owns biological samples that are provided by research participants in biomedical research? (Probe: Why do you think so?)

4. **<u>Knowledge about the H3Africa</u>**
   a. Do you know what the H3Africa is? (If the answer is yes, ask respondents to explain what the H3Africa is about; and if the answer is no, explain what the H3Africa is to respondents)
   b. Please explain the H3Africa requirements on consent and future use of biological samples (If respondents do not know the H3Africa requirements on consent and future use of biological samples, explain the requirements to respondents)

c. What is your opinion on the H3Africa requirements on consent and future use of biological samples? (Probe: why he/she holds such an opinion)

5. Suggestions for improving regulations/guidelines on consent and use of biological samples and data

a. What can be done to improve the national regulations/guidelines on consent and use of biological samples and data in biomedical research?

b. Is there anything that we did not discuss that you would like to mention or talk about?

THANK YOU VERY MUCH FOR YOUR PARTICIPATION!

APPENDIX F: IN-DEPTH INTERVIEW GUIDE FOR POLICY-MAKERS

Title of the study: Stakeholder views on current policies on broad consent and future use of biological samples and data collected in biomedical studies in Malawi and South Africa

Principal Investigator: [redacted]

Co-investigators: [redacted]

1. What are the current regulations on informed consent in biomedical research in this country? (Probe: Please explain the regulations to me. Why did the government decide to come up with these regulations? Were you involved in developing these regulations)?
2. Do the regulations allow researchers to obtain consent from research participants that allows future research on biological samples that are collected from the research participants? (Probe: why do you think the government decided to come up with such regulations? What is your personal opinion on these regulations on consent?)
3. What are your experiences with the implementation of these regulations on informed consent in current biomedical research practice by researchers and REC members? (Prompt: Do you think researchers/REC members understand these regulations? What particular challenges do they encounter/what do they find difficult?)
4. What do you know about funders' requirements for consent? What is your opinion about their consent requirements?
5. What are the current regulations on use of biological samples and data collected in biomedical research in this country? (Probe: Please explain the regulations to me. Why did the government decide to come up with these regulations? Were you involved in developing these regulations?)
6. What are the current regulations on future use of biological samples and data collected in biomedical research? Do the regulations allow unspecified future use of biological samples and data collected in biomedical research?
7. How do you handle complaints from researchers/REC members and funders on national regulations such as these?

8. Do key stakeholders such as researchers, REC members, funders and research participants contribute to the development of national regulations in biomedical research? Has this happened in the past? If yes, please explain how the process unfolded. (Probe: How best should such key stakeholders contribute to the development of national regulations in biomedical research?
9. Is there anything that we did not discuss that you would like to mention or talk about?

THANK YOU VERY MUCH FOR YOUR PARTICIPATION!

APPENDIX G: IN-DEPTH INTERVIEW GUIDE FOR FUNDERS

Title of the study: Stakeholder views on current policies on consent and future use of biological samples and data collected in biomedical studies in Malawi and South Africa

Principal Investigator: [redacted]

Co-Investigators: [redacted]

1. What are the current requirements for consent for researchers that you fund? (Probe: why did you come up with these requirements?)
2. What are your requirements regarding broad consent or do the requirements allow researchers to obtain broad consent from research participants who participate in biomedical research? (Probe: If yes, why do you think so or if no, why not? What is your personal opinion on broad consent?)
3. What are your experiences with the implementation of these requirements on informed consent in current biomedical research practice by researchers and REC members? (Prompt: Do you think researchers/REC members understand these regulations? What particular challenges do they encounter/what do they find difficult?)
4. What are your current requirements for use of biological samples and data collected in biomedical research? (Probe: Why did you decide to come up with these requirements? Were you personally involved in developing these regulations)
5. Do the requirements allow unspecified future use of biological samples and data collected in biomedical research?
6. How do you ensure that key stakeholders such as researchers, REC members and research participants contribute to the development of funders' policies in biomedical research? (Probe: How best should such key stakeholders contribute to the development of such policies in biomedical research?
7. Is there anything that we did not discuss that you would like to mention or talk about?

THANK YOU VERY MUCH FOR YOUR PARTICIPATION!

APPENDIX H: GUIDE FOR FOCUS GROUP DISCUSSIONS WITH RESEARCH PARTICIPANTS IN BIOMEDICAL RESEARCH

Title of the study: Stakeholder views on current policies on consent and future use of biological samples and data collected in biomedical studies in Malawi and South Africa

Principal Investigator: [redacted]

Co-Investigators: [redacted]

1. **Consent used in biomedical research**

A. What does the word "consent' mean to you? (Probe why do researchers seek consent from potential participants before they enrol them in their studies?)

B. How do researchers obtain consent from potential research participants?? (Probe: what kind of information did the researchers provide to you before they sought your permission to participate in the study?

C. There are four types of consent that can be used in research. These are specific consent, blanket consent, multi-layered/tiered consent and broad consent. Among these types of consent, which type of consent do you prefer and why do you prefer this type of consent?

2. **Opinions on the use of broad consent in biomedical research**

Imagine you are being asked to join a study where the researchers want to take your blood and store it. Researchers ask you for broad consent for unspecified future use of that blood for other studies. What do you think about that? Why do you think so?

3. **Opinions on future use of biological samples collected in biomedical research**

a. Why do you think researchers obtain samples such as blood from research participants? (Probe: what do they use the biological samples for?

b. Can you allow researchers to store your biological samples in definitely? Why or why not?) . c. Who owns biological samples that are provided by research participants? [Probe: Why and why not?]

d. What do you think about the future use of biological samples in unspecified research?

4. Suggestions for improving regulations/guidelines on consent and use of biological samples and data

a. What can be done to improve consent processes and use of biological samples in biomedical research?

b. Is there anything that we did not discuss that you would like to mention or talk about?

THANK YOU VERY MUCH FOR YOUR PARTICIPATION!
